# Burden of Osteoarthritis in Pakistan and Its Provinces From 1990 to 2021: Findings From the Global Burden of Disease Study

**DOI:** 10.7759/cureus.90967

**Published:** 2025-08-25

**Authors:** Muhammad Tayyab, Mahmood Ahmad, Razi Mand Shah, Suleman Shah, Ameer Afzal Khan, Rahman Syed, Muhammad Shabir, Anfal Khan, Mohsin Ali, Fazal Syed

**Affiliations:** 1 Department of Trauma and Orthopaedics, Bradford Teaching Hospitals NHS Foundation Trust, Bradford, GBR; 2 Department of Trauma and Orthopaedics, Milton Keynes University Hospital, Milton Keynes, GBR; 3 Department of Orthopaedics, Saidu Group of Teaching Hospitals, Swat, PAK; 4 Department of Nursing, Fatima College of Health Sciences, Al Ain, ARE; 5 Department of Internal Medicine, Saidu Medical College, Swat, PAK; 6 Department of Internal Medicine, Swat Medical College, Swat, PAK; 7 Department of Internal Medicine, Northwest School of Medicine, Peshawar, PAK

**Keywords:** disability-adjusted life years (dalys), global burden of disease (gbd), osteoarthritis, pakistan, prevalence

## Abstract

Background and aim: Osteoarthritis (OA) is a leading cause of chronic disability worldwide, and its burden is increasing sharply in low- and middle-income countries (LMICs) such as Pakistan. This study aimed to assess the temporal trends, site-specific burden, sex-based disparities, and provincial variations in the prevalence, incidence, years lived with disability (YLDs), and disability-adjusted life years (DALYs) due to OA in Pakistan from 1990 to 2021, using data from the Global Burden of Disease (GBD) 2021 study.

Methods: Data were extracted from the GBD 2021 database, encompassing national and subnational estimates. Age-standardized rates of OA prevalence, incidence, DALYs, and YLDs were analyzed for Pakistan and its provinces. Site-specific data for knee, hand, hip, and other forms of OA were also evaluated. Sex-stratified analyses were conducted. Temporal patterns were assessed using Joinpoint regression, with annual percent change (APC) and 95% confidence intervals (CIs) calculated for each segment.

Results: Between 1990 and 2021, the prevalence of OA in Pakistan rose from 2.85 million to 8.49 million. The age-standardized prevalence increased from 4,966 (95% UI: 4,389-5,580) to 5,854 (95% UI: 5,137-6,550) per 100,000 population, a 17.9% rise. Women experienced a higher burden, reaching 7,179 per 100,000 in 2021 versus 4,645 in men. Islamabad Capital Territory (ICT) recorded the highest prevalence (7,160 per 100,000), while Khyber Pakhtunkhwa (KPK) had the lowest (5,606 per 100,000). The age-standardized incidence rate rose from 394.1 (95% UI: 346.6-441.4) to 458.0 (95% UI: 402.5-509.9) per 100,000, with knee OA contributing over 61% of new cases. The DALY rate rose by 19.1%, from 168.5 to 200.6 per 100,000. Women had higher DALYs (248.2) than men (157.2). The highest provincial DALYs were observed in Islamabad (250.7) and the lowest in Khyber Pakhtunkhwa (191.3) in 2021.

Conclusion: The OA burden in Pakistan has grown significantly over the past three decades, especially among women and in urbanized provinces. Public health policies should focus on early diagnosis, fair access to rehabilitation, and promoting healthy lifestyles to reduce the growing burden of osteoarthritis.

## Introduction

Osteoarthritis (OA), often referred to as the “wear and tear” disease, is a chronic joint condition that causes pain, stiffness, and progressive loss of joint function. It primarily affects the knees and hands, is more common in women and older adults, and is strongly linked to modifiable factors, such as a high body mass index (BMI) [1,2]. Common therapeutic approaches for OA include weight management, exercise therapy, analgesics or nonsteroidal anti-inflammatory drugs (NSAIDs), intra-articular injections, and, in severe cases, joint replacement surgery. Globally, the number of people living with OA has more than doubled from about 20.9 million in 1990 to 46.6 million in 2021. This rise has also brought a dramatic increase in years lived with disability (YLDs), making OA one of the leading causes of long-term pain and physical limitation worldwide [3].

In Pakistan, community-based studies have reported OA rates of 14%-20%. Still, these likely represent only the “tip of the iceberg,” as many people with joint pain never reach medical facilities due to cost, distance, or lack of awareness [4]. A population-based study estimated that roughly one in four adults, 28% in urban areas and 25% in rural areas, suffer from knee OA [5]. This disease not only impacts physical health but also disrupts livelihoods, reduces productive years, and stretches already limited healthcare resources [2,6]. Pakistan has been recognized as one of the fastest-growing countries in terms of knee OA burden [6]. Recent analyses show that in South Asia, Pakistan experienced about a 17% rise in age-standardized OA prevalence between 1990 and 2021 [1,7]. Despite these alarming numbers, detailed national and provincial data on OA remain scarce. The Global Burden of Disease (GBD) 2021 dataset now makes it possible to explore OA trends over three decades with breakdowns by age, sex, and sociodemographic index (SDI) [1,7].

Understanding how OA has evolved in Pakistan and how its burden differs between provinces is critical for several reasons. First, it reveals how aging, lifestyle changes, and gender disparities shape OA risk across the country. Second, differences between more industrialized regions like Punjab and rural provinces like Khyber Pakhtunkhwa (KPK) and Balochistan may point to unique risk profiles. Third, tracking changes in incidence, prevalence, and YLDs can help healthcare planners anticipate future needs.

By presenting long-term, province-level data, this study fills a major gap in existing research. It lays the groundwork for more equitable healthcare policies and targeted interventions. Ultimately, mapping the burden of OA over 30 years will help us understand whether prevention efforts, healthcare access, and public awareness have kept pace with this growing challenge. Findings from Pakistan will also contribute to the broader global understanding of OA, particularly within low- and middle-income countries (LMICs) [2,6].

## Materials and methods

Overview

This study utilized data from the Global Burden of Disease (GBD) 2021 study conducted by the Institute for Health Metrics and Evaluation (IHME), the most comprehensive epidemiological assessment of disease burden to date. The GBD 2021 framework provides annual estimates for 371 diseases and injuries across 204 countries and territories, spanning from 1990 to 2021, organized into 21 regions and seven super regions [8,9]. We analyzed data on OA in Pakistan and its subnational units: Punjab, Sindh, Khyber Pakhtunkhwa, Balochistan, Islamabad Capital Territory (ICT), Gilgit-Baltistan (GB), and Azad Jammu and Kashmir (AJK). The methods and modeling frameworks used in the GBD study have been extensively documented in previous publications [8-11]. All data were accessed via the GBD Results Tool and the Global Health Data Exchange (GHDx) platform [12,13].

Case definition and data sources

Osteoarthritis was defined in GBD 2021 as a radiographically confirmed, symptomatic disease with Kellgren-Lawrence (KL) grades 2-4 [8,14]. KL grade 2 was characterized by osteophyte formation and joint pain for at least one month in the preceding year, while grades 3 and 4 involved progressive joint space narrowing [8,15]. Site-specific estimates were generated for knee, hip, hand, and other joint types; spinal OA was excluded as it is classified under low back pain within the GBD framework [8,16].

Data inputs were obtained from a wide range of sources, including national surveys, hospital registries, civil registration systems, and population-based epidemiological studies. Additional data for the South Asian region were derived from peer-reviewed literature and studies such as the Community-Oriented Program for Control of Rheumatic Diseases (COPCORD) [8,10,17]. Studies were included based on standardized criteria, including population representativeness, sample size (≥150), and methodological rigor. Non-population-based studies and reviews were excluded [8].

Estimation of prevalence, incidence, YLDs, and DALYs

Prevalence, incidence, and years lived with disability (YLDs) were estimated using DisMod-MR 2.1, a Bayesian meta-regression tool developed by IHME to synthesize heterogeneous data sources and ensure consistency across disease parameters [8,18]. The model was adjusted for age, sex, location, and study design. Given the non-fatal nature of OA, both remission and mortality rates were set to zero. The model only included individuals aged ≥30 years.

To reduce bias across sources, estimates were corrected using Meta-Regression-Bayesian, Regularized, Trimmed (MR-BRT). Severity levels were categorized using the Western Ontario and McMaster Universities Arthritis Index (WOMAC): mild (0-5), moderate (6-13), and severe (≥14), based on pooled South Asian clinical datasets [8,19]. Disability weights (DWs) were assigned to each severity level using data from household surveys and population panels, ranging from 0 (perfect health) to 1 (death) [8,20]. YLDs were calculated as the product of prevalence and DWs, adjusted for comorbidity overlap using simulation models. Since OA is not a cause of mortality in the GBD framework, disability-adjusted life years (DALYs) are equivalent to YLDs [8].

Data extraction and statistical analysis

We extracted sex-specific, crude, and age-standardized estimates for OA prevalence, incidence, DALYs, and YLDs at the national and subnational levels in Pakistan from 1990 to 2021. Data for individual OA sites (knee, hip, hand, and other) were also extracted. We calculated absolute numbers and percentage changes over time for each metric.

Temporal trends were assessed using Joinpoint regression analysis (Joinpoint Regression Program v5.0.2, National Cancer Institute, Bethesda, MD), which identifies statistically significant changes in trend segments. Annual percent changes (APCs), 95% confidence intervals (CIs), and p-values were reported for each segment.

Ethical considerations

This study involved analysis of publicly available, de-identified secondary data from the GBD database and did not require ethical approval.

**Figure 1:**
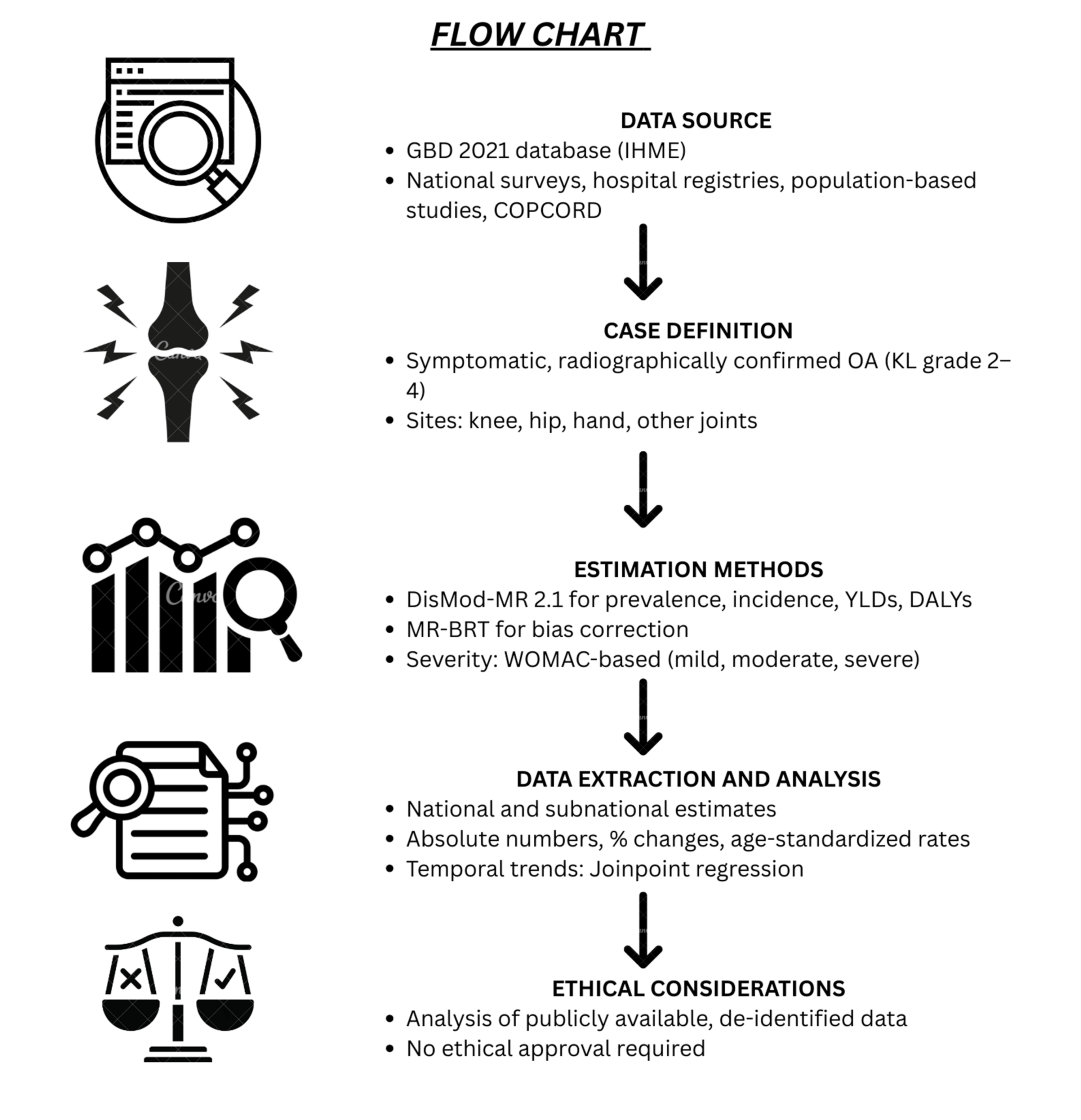
Flowchart of the Study Methodology GBD: Global Burden of Disease, IHME: Institute for Health Metrics and Evaluation, COPCORD: Community-Oriented Program for Control of Rheumatic Diseases, OA: osteoarthritis, KL: Kellgren-Lawrence, YLDs: years lived with disability, DALYs: disability-adjusted life years, MR-BRT: Meta-Regression-Bayesian, Regularized, Trimmed, WOMAC: Western Ontario and McMaster Universities Arthritis Index

Figure 1 presents the flowchart of the study methodology.

## Results

Prevalence

**Figure 2:**
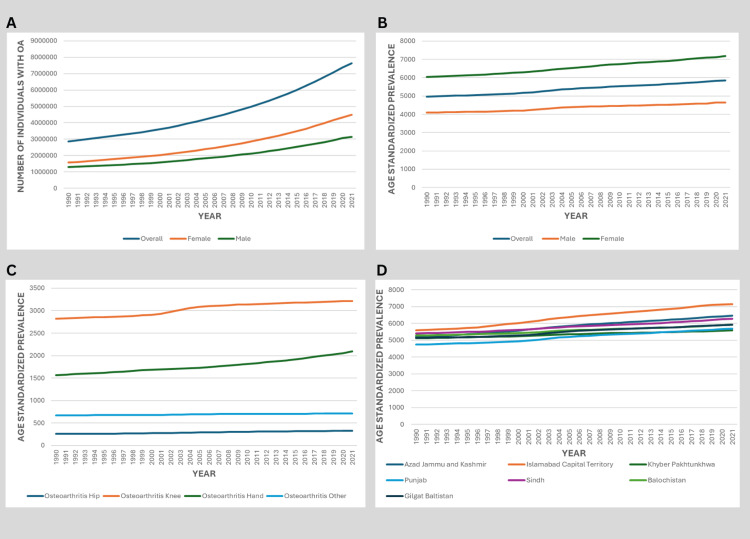
Trends in Osteoarthritis Prevalence in Pakistan by Sex, Joint Site, and Province (1990-2021) A: Absolute number of prevalent OA cases by sex. B: Age-standardized prevalence rate (per 100,000 population) by sex. C: Age-standardized prevalence rate by OA type (knee, hand, hip, and other). D: Provincial distribution of age-standardized OA prevalence in 2021. Data source: GBD 2021 study OA: osteoarthritis, GBD: Global Burden of Disease

Over the last three decades, the prevalence of OA in Pakistan has shown a notable increase. In 1990, the estimated number of OA cases was 2.85 million, increasing to 8.49 million by 2021, as shown in Figure 2A. The age-standardized prevalence of OA per 100,000 persons increased from 4,966 (95% UI: 4,389-5,580) in 1990 to 5,854 (95% UI: 5,137-6,550) in 2021, highlighting a rising burden across all age groups, as shown in Figure 2B.

The age-standardized prevalence in women increased from 6,047 (95% UI: 5,269-6,779) in 1999 to 7,179 (95% UI: 6,215-8,030) in 2021, while in men, it rose from 4,092 (95% UI: 3,624-4,598) in 1999 to 4,645 (95% UI: 4,137-5,240) in 2021, as shown in Figure 2B. For instance, in 2021, the prevalence was 4.49 million among women and 3.12 million among men, as shown in Figure 2A.

In 2021, the age-standardized prevalence of OA demonstrated notable variation across the provinces and territories of Pakistan. The highest prevalence was observed in the Islamabad Capital Territory, with 7,160 (95% UI: 6,338-7,964), followed by Azad Jammu and Kashmir at 6,456 (95% UI: 5,687-7,212). Sindh also reported a relatively high burden of 6,284 (95% UI: 5,567-6,993). Moderate prevalence levels were recorded in Balochistan and Gilgit-Baltistan, with 5,934 (95% UI: 5,277-6,620) and 5,923 (95% UI: 5,239-6,595) cases per 100,000, respectively. Punjab and Khyber Pakhtunkhwa reported comparatively lower prevalence rates, at 5,700 (95% UI: 4,968-6,433) and 5,606 (95% UI: 4,973-6,203), respectively, as shown in Figure 2D.

Between 1990 and 2021, the age-standardized prevalence of different OA types in Pakistan demonstrated a rising trend. Among all types, knee OA consistently exhibited the highest burden. In 1990, the age-standardized prevalence per 100,000 population was estimated at 2,821 (95% UI: 2,349-3,324) for knee OA, 1,566 (95% UI: 1,184-2,014) for hand OA, 254 (95% UI: 195-324) for hip OA, and 669 (95% UI: 522-876) for other OA types. By 2021, these figures had increased to 3,213 (95% UI: 2,693-3,748) for knee OA, 2,098 (95% UI: 1,567-2,683) for hand OA, 327 (95% UI: 249-415) for hip OA, and 711 (95% UI: 552-940) for other OA, as shown in Figure 2C.

Incidence

**Figure 3:**
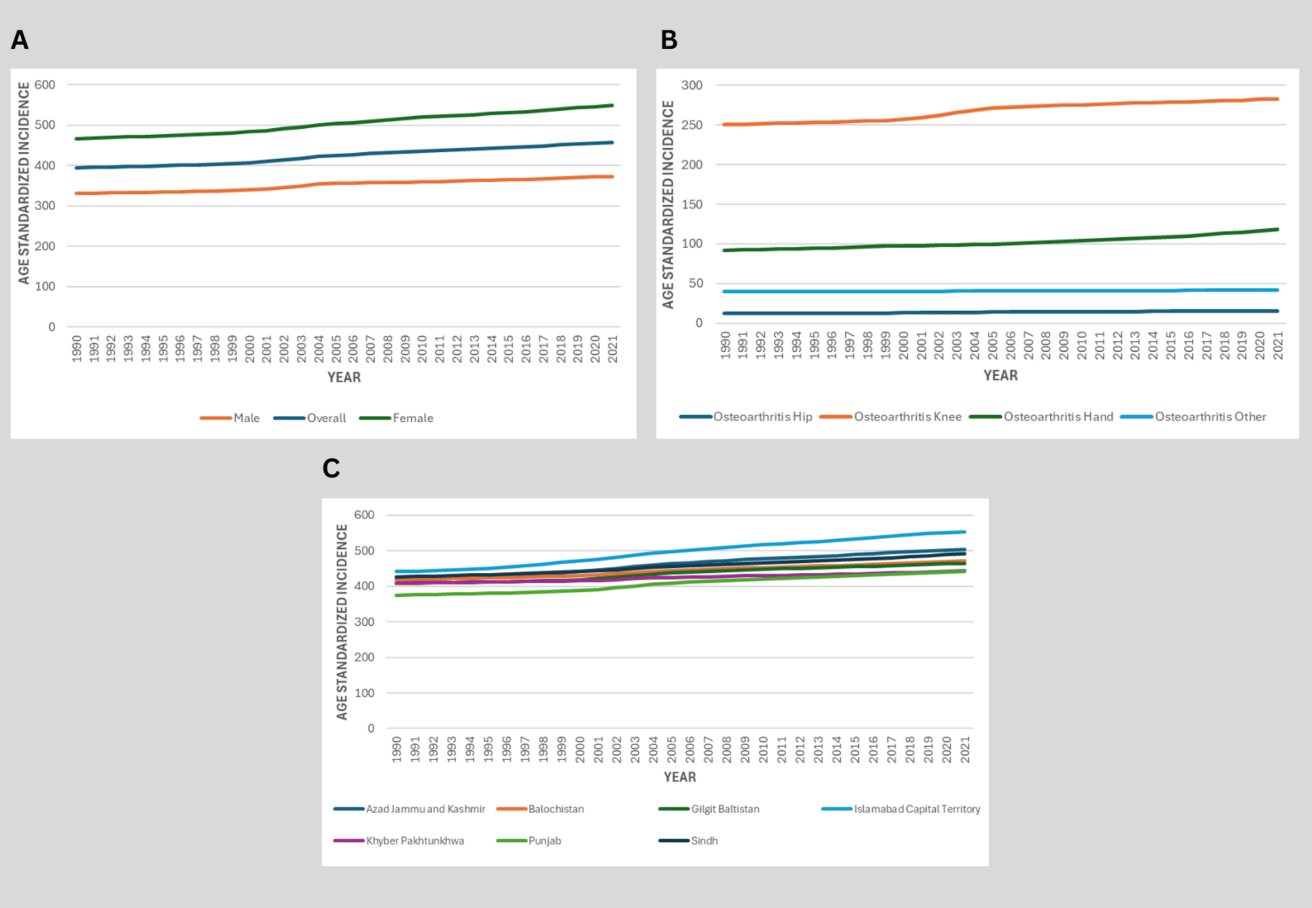
Trends in Osteoarthritis Incidence in Pakistan by Sex, Joint Site, and Province (1990-2021) A: Age-standardized incidence rate by sex. B: Age-standardized incidence rate by OA type (knee, hand, hip, and other). C: Provincial distribution of age-standardized OA incidence in 2021. Data source: GBD 2021 study OA: osteoarthritis, GBD: Global Burden of Disease

The national incidence of osteoarthritis in Pakistan exhibited a sustained increase over three decades, with the annual number of new cases growing from 249,293 (95% UI: 218,772-280,263) in 1990 to 710,195 (95% UI: 618,261-798,896) in 2021, a 2.85-fold increase (185% growth). The overall age-standardized incidence rate increased from 394.1 per 100,000 population (95% UI: 346.6-441.4) in 1990 to 458.0 per 100,000 population (95% UI: 402.5-509.9) in 2021, representing a 16.2% rise. Women consistently showed higher incidence rates than men, with female rates increasing from 466.6 (95% UI: 406.4-522.8) to 548.1 (95% UI: 476.0-613.5), while male rates rose from 330.6 (95% UI: 291.6-371.7) to 373.3 (95% UI: 328.0-418.6), maintaining a stable female-to-male ratio of approximately 1.5:1 throughout the study period, as shown in Figure 3A.

Analysis by affected joints revealed knee OA as the most prevalent form, reaching 282.2 per 100,000 (95% UI: 238.3-326.2) in 2021 and accounting for 61.6% of total osteoarthritis incidence. Hand osteoarthritis demonstrated the steepest relative increase at 28.9% since 1990, rising to 118.4 per 100,000 (95% UI: 88.2-150.4). Hip osteoarthritis showed more modest but steady growth of 28.9% over the same period, reaching 15.8 per 100,000 (95% UI: 11.9-19.9), while other osteoarthritis types remained relatively stable with only a 5.2% increase, as shown in Figure 3B.

The age-standardized incidence of OA in Pakistan has shown a concerning upward trend across all provinces from 1990 to 2021. In 1999, Islamabad Capital Territory (ICT) already had the highest incidence rate at 467.04 per 100,000 population (95% UI: 412.83-518.80), followed by Sindh at 439.33 (95% UI: 389.94-486.99) and Balochistan at 428.89 (95% UI: 381.07-476.97). By 2021, these rates had increased significantly, with ICT reaching 553.19 (95% UI: 487.07-615.37), an 18.4% increase, Sindh at 491.72 (95% UI: 434.82-547.41), a 11.9% rise, and Balochistan at 472.73 (95% UI: 418.74-525.58), a 10.2% increase. Punjab, while having the lowest rates in both years, still experienced substantial growth. The incidence rose from 386.14 (95% UI: 335.25-435.84) in 1999 to 442.91 (95% UI: 385.16-496.97) in 2021, marking a 14.7% increase. Similarly, Khyber Pakhtunkhwa saw its rates climb from 415.22 (95% UI: 366.98-460.25) to 444.47 (95% UI: 394.15-492.96), a 7% rise. The mountainous regions of Azad Jammu and Kashmir and Gilgit-Baltistan showed intermediate patterns, with AJK increasing from 438.89 (95% UI: 391.55-487.72) to 503.82 (95% UI: 445.32-559.35), a 14.8% jump, and GB from 416.92 (95% UI: 370.53-463.96) to 464.46 (95% UI: 410.73-515.27), an 11.4% increase, as shown in Figure 3C.

Disability-adjusted life years (DALYs)

**Figure 4:**
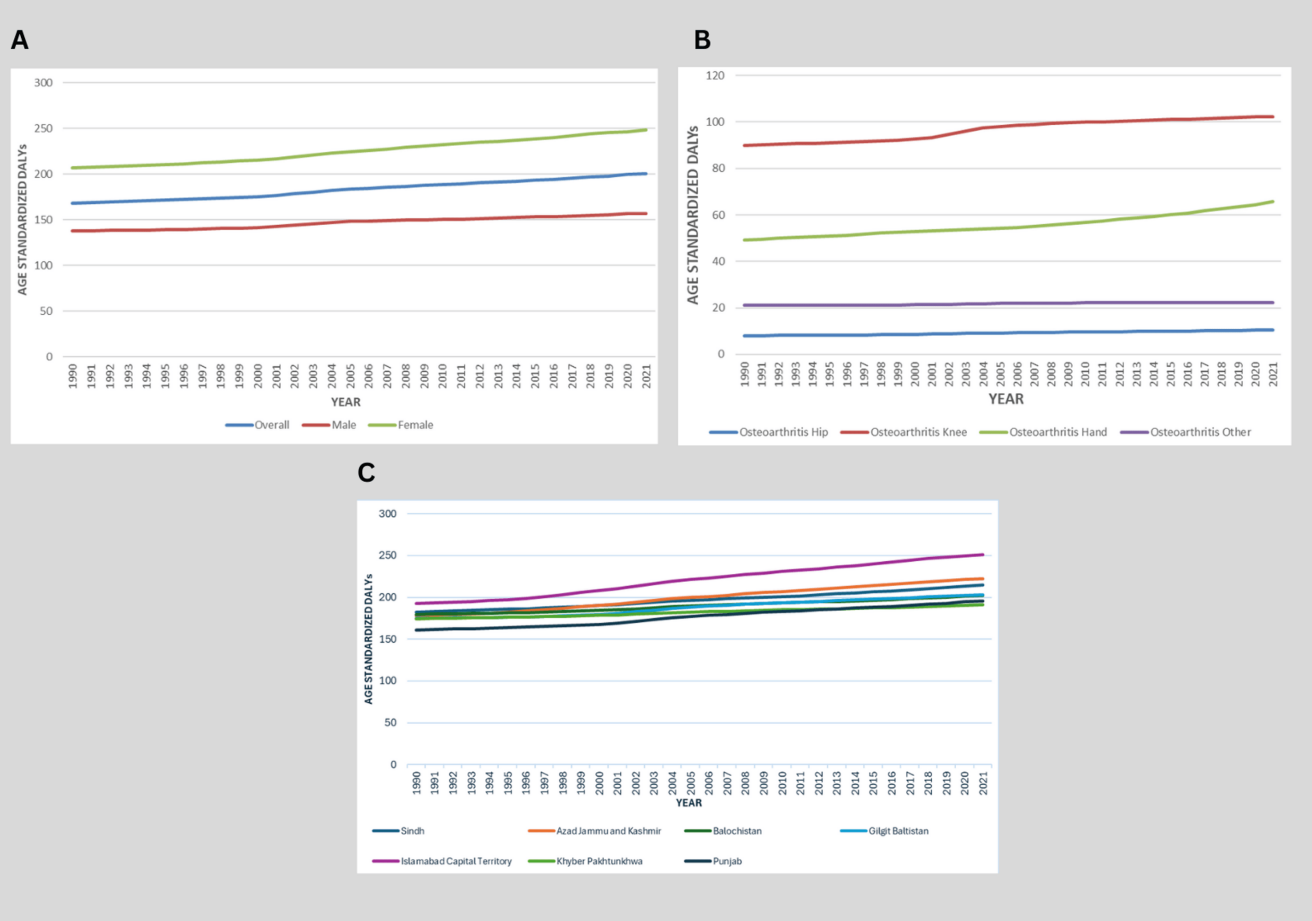
Trends in Osteoarthritis DALYs in Pakistan by Sex, Joint Site, and Province (1990-2021) A: Age-standardized DALY rate by sex. B: Age-standardized DALY rate by OA type (knee, hand, hip, and other). C: Provincial distribution of age-standardized OA DALYs in 2021. Data source: GBD 2021 study DALY: disability-adjusted life year, OA: osteoarthritis, GBD: Global Burden of Disease

From 1990 to 2021, Pakistan experienced a significant and sustained increase in the burden of OA, as measured by age-standardized DALYs. The national DALY rate rose from 168.48 per 100,000 population (95% UI: 79.89-341.15) in 1990 to 200.60 per 100,000 (95% UI: 95.98-403.02) in 2021, marking a 19.1% increase over three decades. Sex disparities were striking, with women bearing a disproportionately higher burden than men. Female DALYs increased from 206.60 per 100,000 (95% UI: 98.41-417.40) in 1990 to 248.18 per 100,000 (95% UI: 118.82-493.38) in 2021 (+20.1%), while male DALYs rose more modestly from 137.78 (95% UI: 66.21-277.98) to 157.22 (95% UI: 75.41-319.09) (+14.1%). By 2021, women accounted for 61.3% of total OA DALYs, as shown in Figure 4A.

When stratified by OA subtype, knee OA remained the leading contributor, with DALYs increasing from 89.99 per 100,000 (95% UI: 44.24-175.78) in 1990 to 102.10 (95% UI: 49.84-199.58) in 2021 (+13.5%). Hand OA exhibited the steepest rise (+33.4%), climbing from 49.30 (95% UI: 22.65-100.37) to 65.74 (95% UI: 30.09-135.01). In contrast, hip OA, although less prevalent, grew by 28.6% (from 8.06 to 10.36 per 100,000), while other OA subtypes remained relatively stable, as shown in Figure 4B.

The age-standardized OA burden showed significant variation across Pakistan’s provinces. Islamabad Capital Territory had the highest 2021 DALY rate at 250.71 per 100,000 population (95% UI: 119.60-500.56), representing a 30% increase from 1990 (192.85; 95% UI: 92.59-393.45). Sindh maintained the second-highest burden in 2021 (214.73; 95% UI: 103.10-428.24), up 17.6% from 1990 (182.58; 95% UI: 87.16-369.03). Azad Jammu and Kashmir demonstrated particularly rapid growth, increasing 25% to 222.31 DALYs/100,000 (95% UI: 105.28-447.52) in 2021 from 177.80 (95% UI: 85.83-362.05) in 1990. Balochistan’s burden rose 12.9% to 202.59 (95% UI: 97.24-405.04) from 179.46 (95% UI: 84.39-361.67), while Gilgit-Baltistan increased 16% to 203.15 (95% UI: 96.83-405.99) from 175.11 (95% UI: 84.91-351.43). Punjab had the lowest 2021 rate at 195.65 DALYs/100,000 (95% UI: 94.87-392.20), although this still represented a 21.7% increase from 160.79 (95% UI: 77.06-325.82) in 1990. Khyber Pakhtunkhwa showed the most modest growth (+9.8%), reaching 191.33 (95% UI: 90.82-386.48) in 2021 compared to 174.29 (95% UI: 83.19-354.18) in 1990, as shown in Figure 4C.

Years lived with disability (YLDs)

**Figure 5:**
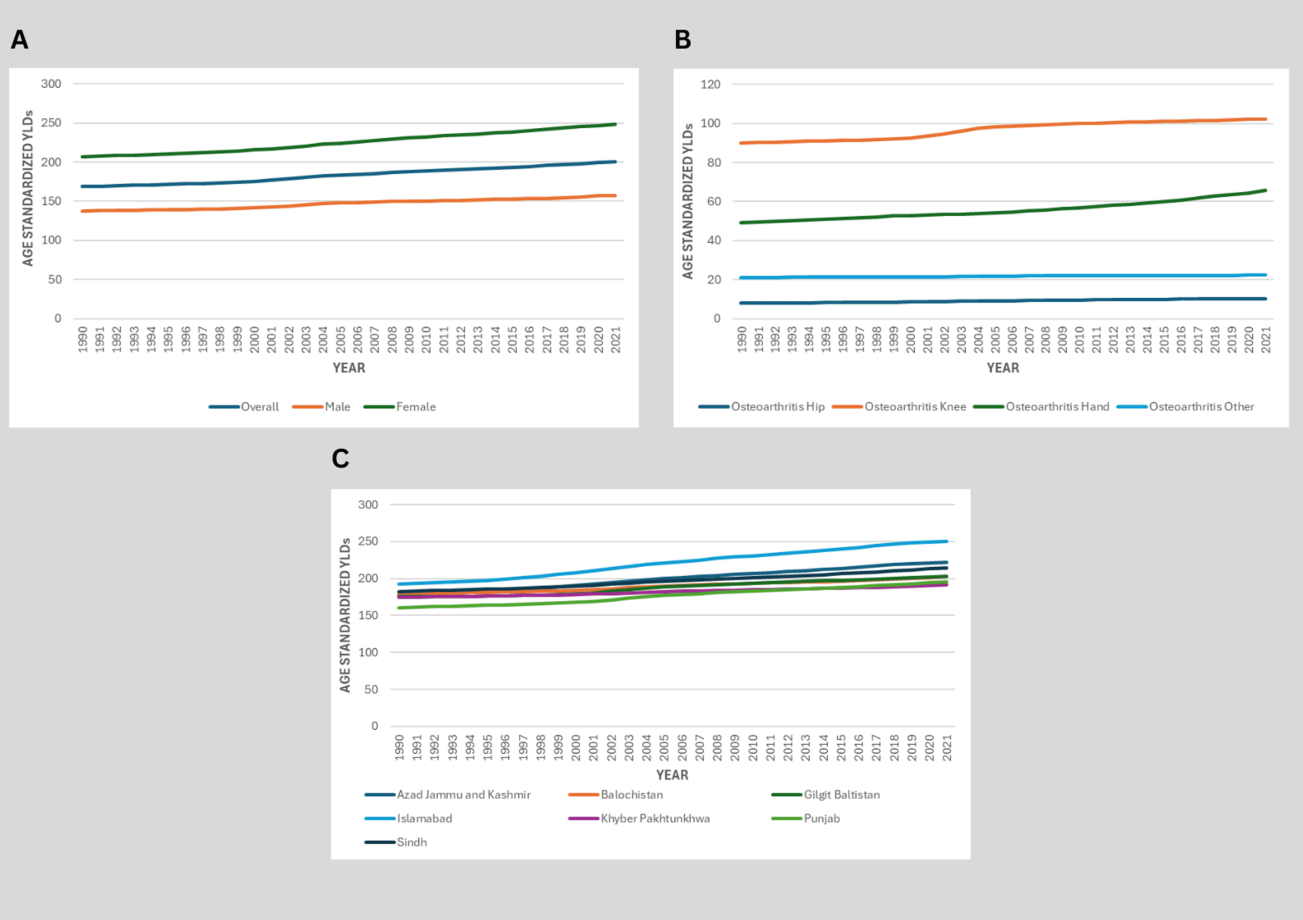
Trends in Osteoarthritis YLDs in Pakistan by Sex, Joint Site, and Province (1990-2021) A: Age-standardized YLD rate by sex. B: Age-standardized YLD rate by OA type (knee, hand, hip, and other). C: Provincial distribution of age-standardized OA YLDs in 2021. Data source: GBD 2021 study YLD: years lived with disability, OA: osteoarthritis, GBD: Global Burden of Disease

The age-standardized YLD rates for OA in Pakistan showed a concerning upward trend between 1990 and 2021. The overall YLD rate increased from 168.48 (95% UI: 79.89-341.15) in 1990 to 200.60 (95% UI: 95.98-403.02) in 2021, representing a 19.1% increase over the 31 years. This rise was particularly pronounced among women, whose YLD rates climbed from 206.60 (95% UI: 98.41-417.40) to 248.18 (95% UI: 118.82-493.38), a 20.1% increase. Men experienced a slightly lower but still significant increase of 14.1%, from 137.78 (95% UI: 66.21-277.98) to 157.22 (95% UI: 75.41-319.09), as shown in Figure 5A.

When examining specific joint sites, knee OA remained the predominant contributor, with YLDs increasing by 13.5% from 89.99 (95% UI: 44.24-175.78) to 102.10 (95% UI: 49.84-199.58). Hand OA showed the most dramatic rise, jumping 33.2% from 49.30 (95% UI: 22.65-100.37) to 65.74 (95% UI: 30.09-135.01). Hip OA YLDs increased by 28.6% from 8.06 (95% UI: 3.75-16.10) to 10.36 (95% UI: 4.79-21.18), while other joint sites saw a more modest 5.9% increase from 21.14 (95% UI: 9.82-44.32) to 22.40 (95% UI: 10.44-47.76), as shown in Figure 5B.

The age-standardized YLD rates for OA varied significantly across Pakistan’s provinces between 1990 and 2021. Islamabad Capital Territory had the highest burden in both years, increasing from 192.85 (95% UI: 92.59-393.45) in 1990 to 250.71 (95% UI: 119.60-500.56) in 2021, reflecting a 30% rise, the largest provincial increase. Balochistan followed, with YLDs growing from 179.46 (95% UI: 84.39-361.67) to 202.59 (95% UI: 97.24-405.04), a 12.9% increase. Sindh showed a steady climb from 182.58 (95% UI: 87.16-369.03) to 214.73 (95% UI: 103.10-428.24), marking a 17.6% rise. In Khyber Pakhtunkhwa (KPK), YLDs increased moderately from 174.29 (95% UI: 83.19-354.18) to 191.33 (95% UI: 90.82-386.48), a 9.8% increase, while Punjab exhibited the lowest baseline rate but a notable 21.7% growth, from 160.79 (95% UI: 77.06-325.82) to 195.65 (95% UI: 94.87-392.20). Gilgit-Baltistan (GB) mirrored this trend, rising from 175.11 (95% UI: 84.91-351.43) to 203.15 (95% UI: 96.83-405.99), a 16% increase, as shown in Figure 5C.

Joinpoint regression analysis

Joinpoint regression analysis revealed significant increases in the burden of osteoarthritis in Pakistan from 1990 to 2021 across all three indicators: prevalence, DALYs, and YLDs. For prevalence, six distinct segments were identified. From 1990 to 1997, the age-standardized prevalence increased at an annual percent change (APC) of 0.35% (95% CI: 0.34-0.36, p < 0.001). From 1997 to 2001, the trend rose more steeply with an APC of 0.54% (95% CI: 0.51-0.58, p < 0.001), followed by the sharpest increase of 1.01% (95% CI: 0.93-1.08, p < 0.001) from 2001 to 2004. Between 2004 and 2008, the APC slowed slightly to 0.56% (95% CI: 0.52-0.59, p < 0.001) and continued at 0.45% (95% CI: 0.44-0.46, p < 0.001) from 2008 to 2016, before rising again to 0.61% (95% CI: 0.59-0.63, p < 0.001) from 2016 to 2021, as shown in Table 1.

**Table 1:** Joinpoint Regression Analysis for Trends in Osteoarthritis From 1990 to 2021 *Significant APC (p < 0.05) APC: annual percent change, CI: confidence interval, DALYs: disability-adjusted life years, YLDs: years lived with disability

Cohort	Segment	Lower endpoint	Upper endpoint	APC	Lower CI	Upper CI	Prob > |t|
Prevalence	1	1990	1997	0.35*	0.34	0.36	<0.001
Prevalence	2	1997	2001	0.54*	0.51	0.58	<0.001
Prevalence	3	2001	2004	1.01*	0.93	1.08	<0.001
Prevalence	4	2004	2008	0.56*	0.52	0.59	<0.001
Prevalence	5	2008	2016	0.45*	0.44	0.46	<0.001
Prevalence	6	2016	2021	0.61*	0.59	0.63	<0.001
DALYs	1	1990	1997	0.36*	0.34	0.37	<0.001
DALYs	2	1997	2001	0.55*	0.5	0.59	<0.001
DALYs	3	2001	2004	1.06*	0.96	1.16	<0.001
DALYs	4	2004	2008	0.59*	0.54	0.64	<0.001
DALYs	5	2008	2016	0.51*	0.49	0.52	<0.001
DALYs	6	2016	2021	0.64*	0.62	0.67	<0.001
YLDs	1	1990	1997	0.36*	0.34	0.37	<0.001
YLDs	2	1997	2001	0.55*	0.5	0.59	<0.001
YLDs	3	2001	2004	1.06*	0.96	1.16	<0.001
YLDs	4	2004	2008	0.59*	0.54	0.64	<0.001
YLDs	5	2008	2016	0.51*	0.49	0.52	<0.001
YLDs	6	2016	2021	0.64*	0.62	0.67	<0.001

For DALYs, the trends closely paralleled those of prevalence. From 1990 to 1997, DALYs increased at an APC of 0.36% (95% CI: 0.34-0.37, p < 0.001), followed by a sharper rise of 0.55% (95% CI: 0.50-0.59, p < 0.001) from 1997 to 2001. The most rapid increase occurred between 2001 and 2004 with an APC of 1.06% (95% CI: 0.96-1.16, p < 0.001), then moderated to 0.59% (95% CI: 0.54-0.64, p < 0.001) from 2004 to 2008, 0.51% (95% CI: 0.49-0.52, p < 0.001) from 2008 to 2016, and finally 0.64% (95% CI: 0.62-0.67, p < 0.001) from 2016 to 2021, as shown in Table 1.

Similarly, YLDs showed identical segment patterns and values to DALYs, highlighting the chronic disabling course of osteoarthritis without substantial fatality. The APC for YLDs was 0.36% (95% CI: 0.34-0.37, p < 0.001) from 1990 to 1997, 0.55% (95% CI: 0.50-0.59, p < 0.001) from 1997 to 2001, 1.06% (95% CI: 0.96-1.16, p < 0.001) from 2001 to 2004, 0.59% (95% CI: 0.54-0.64, p < 0.001) from 2004 to 2008, 0.51% (95% CI: 0.49-0.52, p < 0.001) from 2008 to 2016, and 0.64% (95% CI: 0.62-0.67, p < 0.001) from 2016 to 2021, as shown in Table 1.

## Discussion

This national-level analysis of OA trends in Pakistan from 1990 to 2021 using the GBD framework reveals a substantial increase in the burden of OA across all key metrics: prevalence, incidence, DALYs, and YLDs. Our study reveals not only the growing prevalence of OA but also significant differences by gender, joint subtype, and geographic region.

The findings indicate that the age-standardized prevalence of OA rose by 17.8% over the 31 years, while incidence increased by 16.2%, YLDs by 19.1%, and DALYs by 19.1%. These increases are consistent with global patterns of OA burden escalation, notably in low- and middle-income countries (LMICs) experiencing demographic transitions such as population ageing and rising obesity rates [3,15]. Consistent with worldwide evidence, women had a much larger burden than men across all parameters [21]. Biological susceptibility, hormonal factors, and healthcare-seeking behavior may all contribute to the gender disparity [21,22].

Among joint-specific OA subtypes, knee OA remained the most prevalent and burdensome, accounting for over 60% of cases and the highest share of DALYs and YLDs in 2021. Hand OA, although less disabling, showed the greatest relative increase in both prevalence and DALYs, probably due to increased diagnosis or lifestyle changes such as occupational hand use [23]. Hip OA, on the other hand, remained relatively uncommon in Pakistan, keeping with previous regional evidence indicating that lower biomechanical loading patterns and genetic predispositions may minimize its prevalence when compared to Western populations [24].

From a geographic aspect, the Islamabad Capital Territory (ICT) consistently had the highest age-standardized prevalence, incidence, DALYs, and YLDs. This may be due to improved case diagnosis and urban lifestyle risk factors such as physical inactivity, poor diet, and increased obesity prevalence [25]. In contrast, provinces such as Punjab and Khyber Pakhtunkhwa had considerably lower OA indicators, which could be attributed to a combination of underreporting, poor healthcare access, and a potentially younger population demographic [26].

Our Joinpoint regression analysis offers valuable insights into temporal dynamics. The most notable annual percent changes (APCs) in prevalence, DALYs, and YLDs occurred between 2001 and 2004, corresponding with epidemiological shifts, increased urbanization, and changes in healthcare utilization [3,27]. The most recent interval (2016-2021) also showed a continued acceleration in OA burden (prevalence APC: 0.61%, DALY APC: 0.64%, YLD APC: 0.64%), which may reflect increased obesity and ageing trends [25,28].

The rising OA load in Pakistan poses major difficulties to public health systems. Despite being non-fatal, OA causes severe disability, diminished quality of life, and loss of economic output [29]. The disproportionate impact on women needs gender-sensitive preventative and treatment plans. Knee osteoarthritis, in particular, should be the focus of national musculoskeletal health programs, such as public education, weight management campaigns, and early physical therapy interventions [22]. There is also a need to strengthen primary care services to facilitate early diagnosis and non-surgical management. Given the relatively low levels of orthopedic surgical capacity and joint replacement accessibility in Pakistan, preventive care and rehabilitation services are crucial to reduce disability and maintain function [30].

This study has several limitations. First, it is based on modelled estimates from the GBD database, which may bring inherent uncertainty and biases, particularly in countries like Pakistan with inadequate high-quality surveillance data. The quality of OA diagnosis and reporting varies greatly across locations and time, influencing trend reliability. Second, OA is typically underdiagnosed, especially in rural and low-resource areas with inadequate healthcare access and diagnostic imaging. As a result, the actual burden could be higher than indicated. Third, the GBD technique fails to capture disease severity or functional outcomes, which are critical for assessing the full societal impact of OA. Fourth, joint-specific OA estimates may lack local validation, given the scarcity of national-level registry data on different OA types. Lastly, we did not assess risk factor trends (e.g., obesity, physical inactivity, and prior joint trauma), which would have enhanced causal interpretations and policy relevance.

## Conclusions

The burden of osteoarthritis in Pakistan has increased substantially over the past three decades, driven by demographic, lifestyle, and epidemiological transitions. The rising trend in all indicators, especially among women and for knee OA, signals the urgent need for comprehensive musculoskeletal health policies and resource allocation. Future research should focus on strengthening national OA surveillance, integrating OA care into primary health systems, and addressing modifiable risk factors to reduce long-term disability and economic costs.
